# A biospectroscopic analysis of human prostate tissue obtained from different time periods points to a trans-generational alteration in spectral phenotype

**DOI:** 10.1038/srep13465

**Published:** 2015-08-27

**Authors:** Georgios Theophilou, Kássio M. G. Lima, Matthew Briggs, Pierre L. Martin-Hirsch, Helen F. Stringfellow, Francis L. Martin

**Affiliations:** 1 Centre for Biophotonics, LEC, Lancaster University, Lancaster LA1 4YQ, UK; 2 Department of Obstetrics and Gynaecology, Central Lancashire Teaching Hospitals NHS Foundation Trust, Preston, UK; 3 Institute of Chemistry, Biological Chemistry and Chemometrics, Federal University of Rio Grande do Norte, Natal 59072-970, RN-Brazil

## Abstract

Prostate cancer is the most commonly-diagnosed malignancy in males worldwide; however, there is marked geographic variation in incidence that may be associated with a Westernised lifestyle. We set out to determine whether attenuated total reflection Fourier-transform infrared (ATR-FTIR) or Raman spectroscopy combined with principal component analysis-linear discriminant analysis or variable selection techniques employing genetic algorithm or successive projection algorithm could be utilised to explore differences between prostate tissues from differing years. In total, 156 prostate tissues from transurethral resection of the prostate procedures for benign prostatic hyperplasia from 1983 to 2013 were collected. These were distributed to form seven categories: 1983–1984 (*n* = 20), 1988–1989 (*n* = 25), 1993–1994 (*n* = 21), 1998–1999 (*n* = 21), 2003–2004 (*n* = 21), 2008–2009 (*n* = 20) and 2012–2013 (*n* = 21). Ten-μm-thick tissue sections were floated onto Low-E (IR-reflective) slides for ATR-FTIR or Raman spectroscopy. The prostate tissue spectral phenotype altered in a temporal fashion. Examination of the two categories that are at least one generation (30 years) apart indicated highly-significant segregation, especially in spectral regions containing DNA and RNA bands (≈1,000–1,490 cm^−1^). This may point towards alterations that have occurred through genotoxicity or through epigenetic modifications. Immunohistochemical studies for global DNA methylation supported this. This study points to a trans-generational phenotypic change in human prostate.

Prostate cancer (PCa) is the most commonly diagnosed male malignancy in the world with an incidence rate of 214 cases per 100,000 and a mortality rate from associated metastatic disease of 30 in 100,000^1^,^2^. The percentage of PCa amongst all male cancers is much higher in developed countries (15%) than in developing ones (4%), but there are also large regional differences in incidence rates^3^,^4^,^5^. The only established risk factors for PCa are increasing age, ethnic origin and heredity^6^,^7^,^8^. However, the effects of environment and lifestyle appear to be important towards its development^9^,^10^. The age-adjusted incidence trends for PCa in the 20-y period from 1973 to 1992 were found to increase consistently in 15 countries^11^. Associated temporal lifestyle variations may include diet and exercise, with related factors in the prevalence of obesity, diabetes and metabolic syndromes, tobacco smoking and alcohol intake^12^,^13^,^14^,^15^,^16^,^17^,^18^,^19^.

Working on the assumption that lifestyle changes are major players in the initiation and development of PCa and that habits, especially dietary, have changed dramatically in the past 20 y (within one generation), we set out to explore differences that may exist between prostates from different individuals obtained over a 30-y period. Tissue from transurethral resection of the prostate (TURP) procedures for benign prostatic hyperplasia (BPH) provided the opportunity to study these temporal differences (Fig. 1). The cancer risk in this population is comparable or marginally increased relative to the general population^20^,^21^. Although prostate tissue from TURP procedures may be histologically benign, it could harbour early molecular alterations that contribute to PCa development.

In the search for such molecular alterations, biospectroscopy may play an important role as it can identify structural alterations of cellular molecules based on chemical bonds^22^,^23^,^24^,^25^. Recent studies have also examined its potential in identifying biomarkers for cancer screening^26^,^27^,^28^. Attenuated total reflection Fourier-transform infrared spectroscopy (ATR-FTIR) and Raman spectroscopy were used to interrogate prostate tissue. The resulting spectral data were analyzed using multivariate analysis in the form of principal component analysis followed by linear discriminant analysis (PCA-LDA) and variable selection techniques in the form of sequential progression algorithm (SPA) or genetic analysis (GA), again followed by LDA (SPA-LDA, GA-LDA). Currently there is a lack of research evaluating potential prostate molecular changes that have occurred in the past 30 y (>1 generation). This study set out to determine if spectral differences in prostate tissue of men of similar ages have occurred from the 1980’s to the present day. This could lend insights into distinct associations between modern adopted lifestyle and risk of PCa.

## Methods

### Tissue collection

Archival benign prostate tissue specimens from TURP procedures were collected from one centre. All experimental protocols herein for the use of archival genitourinary tissue retrieved from the Royal Preston Hospital Research Tissue Bank were approved by the UK National Research Ethics Service (http://www.hra.nhs.uk/about-the-hra/our-committees/nres/; Research Ethics Committee reference: 10/H0308/75). In addition, all the methods carried out in this study were in accordance with the approved guidelines. Informed consent was obtained from 2010 when the biobank started working but the archival tissue is also included in the ethics approval document. They comprised of prostate tissue chippings that were formalin-fixed, dehydrated and paraffin-embedded (FFPE) in pathology blocks (*n* = 156). These specimens were matched for age between sixty and sixty-nine years old. They were also matched for ethnicity with all being “British Caucasian”. All the specimens were examined using routine histopathology procedures and found to be free from PCa and other abnormalities other than BPH. As far as we can ascertain, no significant changes in fixation or paraffin embedding occurred during this time period, and no degradation of tissue architecture was observed.

**Figure 1:**
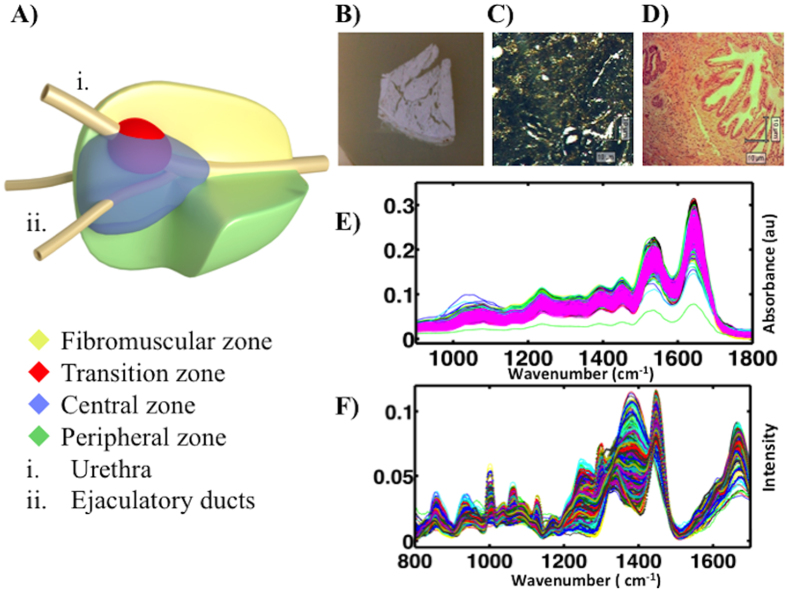
Prostate anatomy, sample preparation and ATR-FTIR or Raman spectroscopy. (**A**) Prostate anatomy illustrating the different histological zones. TURP removes part of the transition zone. (**B**) Low-E slide containing a prepared sample. (**C**) Micrograph of a prostate sample as visualised during Raman spectroscopy. (**D**) H&E stained section for histological comparison and to ensure no diathermy artefacts contaminate the sample. (**E**) Unprocessed ATR-FTIR spectral dataset (*x*-axis: wavenumbers (cm^−1^), *y*-axis: absorbance) (**F**) Unprocessed Raman spectral dataset. (*x*-axis: wavenumbers (cm^−1^), *y*-axis: intensity).

In total, 156 specimens were collected from 1983 to 2013. These samples were distributed according to the year of collection to form seven categories: 1983–1984 (*n* = 20), 1988–1989 (*n* = 25), 1993–1994 (*n* = 21), 1998–1999 (*n* = 21), 2003–2004 (*n* = 21), 2008–2009 (*n* = 20) and 2012–2013 (*n* = 21). Ten-μm-thick tissue sections were floated onto Low-E IR reflective slides (Kevley Technologies, Chesterland, OH, USA) slides for ATR-FTIR spectroscopy. These were de-waxed by serial immersion in three sequential fresh xylenes baths for 5 min and washed in an acetone bath for another 5 min^29^. The resulting samples were allowed to air-dry and then placed in a desiccator until analysis (Fig. 1B,C). Parallel H&E sections were obtained for histological comparison to ensure relevant areas were examined (Fig. 1D).

### ATR-FTIR spectroscopy

IR spectra were obtained using a Bruker Vector 27 FTIR spectrometer with a Helios ATR attachment containing a diamond crystal (≈250 μm × 250 μm sampling area) (Bruker Optics Ltd., Coventry, UK). Spectra were acquired from 10 different locations across each specimen with a new background taken for every new sample. The ATR crystal was cleaned with distilled water and dried with dry tissue paper before the acquisition of spectral background. The spectral resolution was 8 cm^−1^ giving data spacing of 4 cm^−1^. Spectra were co-added for 32 scans; these were converted into absorbance by Bruker OPUS software (Fig. 1E).

### Raman spectroscopy

Raman spectra were acquired using an InVia Renishaw Raman spectrometer (Renishaw plc, Gloucestershire, UK). Its laser diode, operating at 35 mW, emits a mid-IR beam, whose exact wavelength is 785 nm. This was passed through a Rayleigh holographic edge filter. The spectrometer’s entrance slit of 50 mm combined with a diffraction grating of 1,200 lines per mm achieved a spectral resolution of 1 cm^−1^. Raman scatter signals were directed onto a Master Renishaw Pelletier cooled charged couple detector (CCD). Spectra were acquired using a Leica microscope *via* a ×50 objective lens with a numerical aperture of 0.75, giving a spatial resolution of approximately 1 μm. A white light camera mounted on the microscope allowed the use of dark-field visualization of the locations of interest. The Renishaw system was calibrated with a Renishaw silicon calibration source for wavenumber shifts every time the spectrometer was turned on. Ten spectra were acquired from independent locations from each sample. A total of 1,437 spectra were acquired using 100% laser power with an exposure period of 25 seconds and four repeat acquisitions (Fig. 1F). Raman spectroscopy was always performed on the same tissue sections following ATR-FTIR spectroscopy. Independent regions were targeted to minimize any confounding influences due to tissue compression by the ATR crystal.

### Computational analysis

The importing and pre-treatment of the spectral data and the construction of chemometric classification models were executed using PLS toolbox 7.8 (Eigenvector Research, Inc. 3905 West Eaglerock Drive, Wenatchee, WA 98801) and in-house written scripts (IRootLab)^30^ within a MATLAB R2013a environment (Mathworks Inc, Natick, MA, USA).

ATR-FTIR spectra were cut to include wavelengths between 1,800 and 900 cm^−1^ (235 wavenumbers at 3.84 cm^−1^ spectral resolution); the area associated with the biological spectral fingerprints. The resulting dataset was rubber band baseline-corrected and normalized to the Amide I peak (*i.e*., ≈1,650 cm^−1^)^29^,^31^.

Raman spectra contained cosmic rays, which were removed using an in-house tool for Matlab. This algorithm excluded cosmic rays by statistically evaluating the whole spectral dataset (all samples) to identify abnormally high ‘spikes’ that did not present repeatedly. The spectral areas containing these spikes were replaced by appropriate values calculated as a function of intensities for the concerned areas for the rest of the data. The abrangence factor (k = 5) was adjusted to increase the sensitivity of the tool for spike removal. The resulting spectra were cut to include 1,750–800 cm^−1^ (692 data points). Subtraction of biological tissue auto-fluorescence was carried out using an automatic baseline correction method (Whittaker filter)^31^.

Computational analysis consisted of three models: PCA, SPA or GA. All models were followed by LDA^29^. Before applying each analytical model, spectral data were divided into training (70%), validation (15%) and prediction (15%) sets by applying the classic Kennard-Stone (KS) uniform sampling algorithm^32^. The number of samples colonising each set is presented in Electronic Supplementary Information (ESI) Tables S1 and S2 for ATR-FTIR and Raman spectroscopy, respectively. The training datasets were used in the modelling procedures (including variable selection for LDA), whereas the prediction dataset was only used for the final classification evaluation. The optimum number of variables for SPA-LDA and GA-LDA was determined from the minimum cost function G calculated for a given validation dataset:


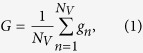


where *g*_*n*_ is defined as


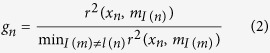


and *I*(*n*) is the index of the true class for the n^th^ validation object *x*_*n*_.

PCA is a multivariate analysis technique that aims to reduce the number of variables present in the spectral dataset. Principal components (PCs) can capture most of the variance (>95%) present in the original dataset. A power *versus* cost calculation identifies the number of PCs that correctly identifies variance within the dataset without presenting artificial separation between the different classes. This optimum number was applied to classify the prostates depending on the year they were excised.

SPA is a forward selection method^33^. Its purpose is to select wavelengths whose information content is minimally redundant to solve co-linearity problems. The model starts with one wavelength, then incorporates a new one at each iteration until it reaches a specified number N of wavelengths^34^. SPA does not modify the original data vectors as PCA does. In this case projections are used only for selection purposes. Thus, the relation between spectral variables and data vectors is preserved.

Genetic algorithms (GA) are combinational algorithms inspired by Mendelian genetics. They use a combination of selection, recombination and mutation to evolve a solution to a problem. They treat data as chromosomes allocating reproductive opportunities in such a way that those chromosomes, which represent a better solution to the target problem are given more chances to “reproduce” than those, which represent poorer solutions^35^. The GA routine was carried out utilising 100 generations containing 200 chromosomes each. Crossover and mutation probabilities were set to 60% and 10%, respectively. Moreover, the algorithm was repeated three times, starting from different random initial populations. The best solution (in terms of the fitness value) resulting from the three realizations of the GA was employed.

LDA was performed following the application of each of the analytical models. LDA scores, loadings, and discriminant function (DF) values were obtained. Usually, the first LDA factor (LD1) is used to visualize the main biochemical alterations within the sample on a 1-dimensional (D) scores plot.

### Immunohistochemistry

Four-μm-thick, parallel sections of prostate specimens from the 1983–1984 (*n* = 10) and the 2012–2013 (*n* = 10) classes were de-waxed in xylene and taken to absolute alcohol. They were then placed in a warm *Tris/EDTA* (Trizma Base, Sigma, T1503; citric acid crystals, BDH277804L; sodium hydroxide, BDH301675N) buffer and heated under pressure at 900 W in a microwave for 4 min. They were then cooled under running water and rinsed with *Tris* buffer. Then, they were treated with hydrogen peroxide blocking agent (*Dako*) for five min, drained and rinsed with *Tris* buffer. Normal blocking serum was then placed on the sections for 20 min followed by 5-methylcytosine as the primary antiserum (5-mc antibody, dilution 1:400; *Genetext: GT4111*) for 60 min. They were then rinsed with *Tris* buffer before adding the secondary antibody (*Vectastain Universal Elite* ABC Kit) for 30 min. After another wash with *Tris* buffer, they were incubated in Strept-ABComplex/HRP (*Vectastain Elite* ABC Reagent) solution for 30 min and then washed again. One drop of chromogen was added to 1 ml of *Dako* and placed on the sections for 10 min. They were then washed under running water before counterstaining with haematoxylin for 5 min, dehydrated in alcohol, cleared in xylene and mounted in styrolite.

## Results

### ATR-FTIR spectral dataset

**Figure 2:**
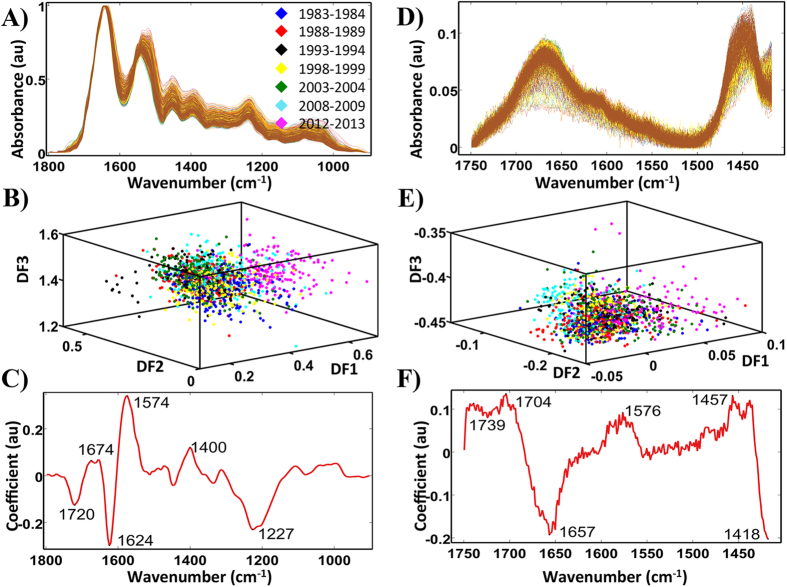
Processing of the ATR-FTIR and Raman derived spectral datasets for all categories by PCA-LDA. (**A**) Pre-processed ATR-FTIR spectral dataset. (**B**) Scores (DF1 × DF2 × DF3) plot calculated by PCA-LDA. (**C**) Loadings plot derived from PCA-LDA. (**D**) Prepossessed Raman spectral dataset. (**E**) Scores (DF1 × DF2 × DF3) plot calculated by PCA-LDA. (**F**) Loadings plot derived from PCA-LDA.

Figure 2A shows the pre-processed ATR-FTIR-derived spectra for prostate chippings according to the year they were collected, generating seven categories: **[1]** 1983–1984 (*n* = 20); **[2]** 1988–1989 (*n* = 25); **[3]** 1993–1994 (*n* = 21); **[4]** 1998–1999 (*n* = 21); **[5]** 2003–2004 (*n* = 21); **[6]** 2008–2009 (*n* = 20); and, **[7]** 2012–2013 (*n* = 21). There is significant overlap between categories and visual inspection alone is limited with regards to identifying distinguishing features. In order to attempt classification of the prostate samples according to year of collection and to determine the biochemical markers responsible for any such classification, it is necessary to apply chemometric analysis techniques. PCA-LDA, SPA-LDA and GA-LDA were therefore adopted to systematically identify spectral differences between the pre-assigned categories.

Figure 2B shows a scores plot derived following PCA-LDA of the ATR-FTIR spectra. This model was carried out using the first six PCs, which account for >90% of the variance within the sample population. Scores plots identify the similarities and dissimilarities between different categories and present them as clusters of points. Loadings plots identify the distinguishing wavenumbers (as weightings). It is obvious that most spectral classes form a single cluster. It is also obvious that there is separation between the 1983–1984 (blue) and the 2012–2013 (pink) categories, which are >one generation (30 y) apart. This separation is significant (*P* < 0.0001). The loadings plot (Fig. 2C) derived from PCA-LDA identifies the six primary wavenumbers, which are important for separation of the different age groups. These include 1,227, 1,400, 1,574, 1,624, 1,674 and 1,720 cm^−1^. ESI: Table S3 lists the molecular entities associated with these wavenumbers.

**Figure 3:**
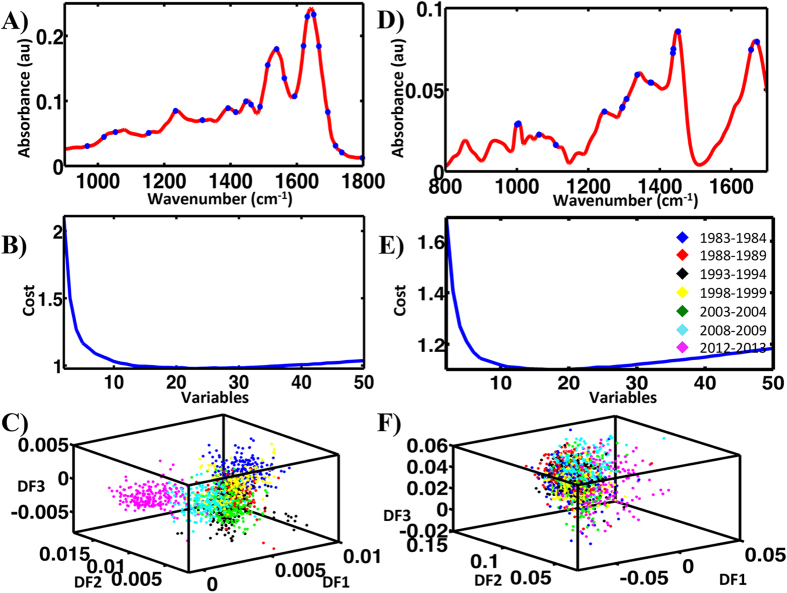
Processing of the ATR-FTIR and Raman spectral datasets for all categories by SPA-LDA. (**A**) Twenty three wavenumbers selected by the SPA-LDA model for the ATR-FTIR spectral dataset. (**B**) Graph representing the power calculation used to identify the optimum number of wavelengths used for SPA. (**C**) Scores (DF1 × DF2 × DF3) plot calculated by SPA-LDA. (**D**) Seventeen wavenumbers selected by SPA-LDA model for the Raman spectral dataset. (**E**) Graph representing the power calculation used to identify the optimum number of wavelengths to be used for SPA. (**F**) Scores (DF1 × DF2 × DF3) plot calculated by SPA-LDA.

SPA-LDA was applied to the dataset using the optimum number of variables derived by identifying the minimum cost from function G (Fig. 3B). The twenty-three wavenumbers selected were: 968, 1,018, 1,053, 1,153, 1,234, 1,315, 1,392, 1,415, 1,446, 1,462, 1,489, 1,512, 1,539, 1,562, 1,593, 1,620, 1,631, 1,651, 1,666, 1,693, 1,716, 1,735 and 1,797 cm^−1^ [Fig. 3A; see ESI Table S4]. The resulting 3-D scores plot (Fig. 3C) identified significant segregation between categories (*P* < 0.05). Spectral points from the same category tend to co-cluster and differing classes segregate. There is a clear progression with time, with categories separated by one generation being furthest apart.

**Figure 4:**
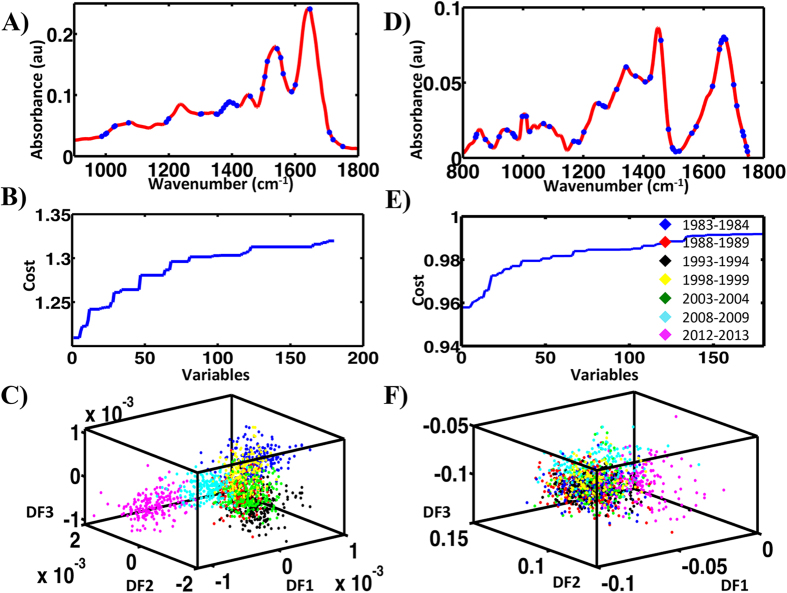
Processing of the ATR-FTIR and Raman datasets for all classes by GA-LDA. (**A**) Thirty two wavenumbers selected by GA-LDA model for the ATR-FTIR dataset. (**B**) Graph representing the power calculation used to identify the optimum number of wavelengths to be used for SPA. (**C**) Scores (DF1 × DF2 × DF3) plot calculated by using the variables selected by GA-LDA from ATR-FTIR spectra obtained from prostate tissues segregated into seven categories. (**D**) Forty nine wavenumbers selected by GA-LDA model for the Raman spectral dataset. (**E**) Graph representing the power calculation used to identify the optimum number of wavelengths to be used for SPA. (**F**) Scores (DF1 × DF2 × DF3) plot calculated by using the variables selected by GA-LDA.

Figure 4C displays the scores plot for classification achieved utilising GA-LDA. The GA model was built based on the selection of 32 wavenumbers (Fig. 4A; see ESI Table S5) out of the available 234, determined by function G (Fig. 4B). These included wavenumbers: 987, 999, 1,002, 1,026, 1,029, 1,072, 1,191, 1,199, 1,299, 1,303, 1,350, 1,353, 1,365, 1,373, 1,381, 1,388, 1,392, 1,404, 1,415, 1,458, 1,496, 1,504, 1,512, 1,543, 1,554, 1,562, 1,589, 1,600, 1,647, 1,708, 1,720 and 1,751 cm^−1^. Again, there is separation between the different categories that is significant (*P* < 0.05).

**Figure 5:**
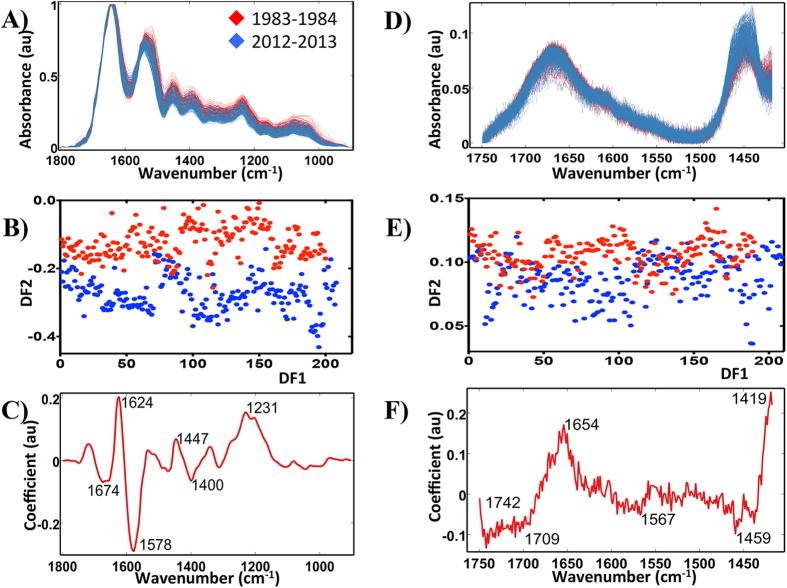
Processing by PCA-LDA of the ATR-FTIR and Raman spectral datasets for categories: 1983–1984 and 2012–2013. (**A**) Pre-processed ATR-FTIR spectral dataset. (**B**) Scores (DF1 × DF2) plot calculated by PCA-LDA. (**C**) Loadings plot derived from PCA-LDA. (**D**) Prepossessed Raman spectral dataset. (**E**) Scores (DF1 × DF2) plot calculated by PCA-LDA. (**F**) Loadings plot derived from PCA-LDA.

When comparing the two categories separated by 28 y (1983–1984 and 2012–2013), the distinction between them is much clearer. Figure 5A shows the pre-processed ATR-FTIR spectra used for analysis applying the three previously mentioned techniques. The 2-D scores plot derived from PCA-LDA of these two categories identifies significant segregation between them (*P* < 0.0001) (Fig. 5B). The associated loadings plot (Fig. 5C) identifies the 6 principal segregating wavenumbers. The molecular entities assigned to these are listed in ESI Table S6.

**Figure 6:**
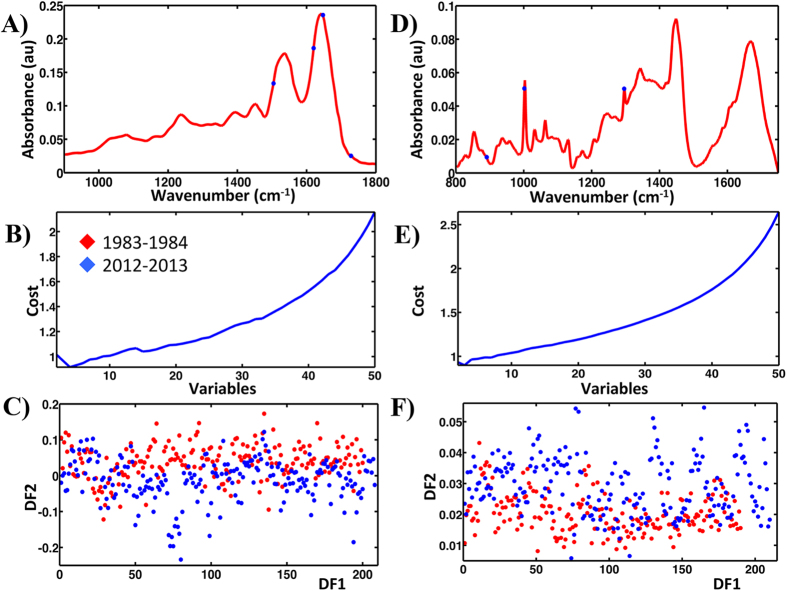
Processing by SPA-LDA of the ATR-FTIR and Raman spectral datasets for categories: 1983–1984 and 2012–2013. (**A**) Four wavenumbers selected by the SPA-LDA model for the ATR-FTIR dataset. (**B**) Graph representing the power calculation used to identify the optimum number of wavelengths used for SPA. (**C**) Scores (DF1 × DF2) plot calculated by SPA-LDA. (**D**) Three wavenumbers selected by SPA-LDA model for the Raman spectral dataset. (**E**) Graph representing the power calculation used to identify the optimum number of wavelengths to be used for SPA. (**F**) Scores (DF1 × DF2) plot calculated by SPA-LDA.

**Figure 7:**
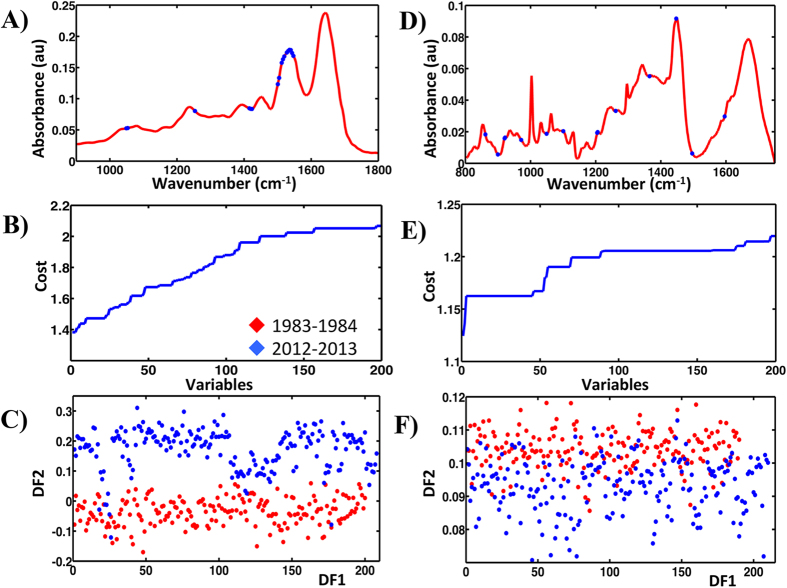
Processing by GA-LDA of the ATR-FTIR and Raman spectral datasets for categories: 1983–1984 and 2012–2013. (**A**) Seventeen wavenumbers selected by GA-LDA model for the ATR-FTIR spectral dataset. (**B**) Graph representing the power calculation used to identify the optimum number of wavelengths to be used for SPA. (**C**) Scores (DF1 × DF2) plot calculated by using the variables selected by GA-LDA from ATR-FTIR spectra obtained from prostate tissues segregated into seven classes. (**D**) Fourteen wavenumbers selected by GA-LDA model for the Raman spectral dataset. (**E**) Graph representing the power calculation used to identify the optimum number of wavelengths to be used for SPA. (**F**) Scores (DF1 × DF2) plot calculated by using the variables selected by GA-LDA.

Similarly SPA-LDA identified significant separation (*P* < 0.05) between the two categories as shown by the related scores plot (Fig. 6C). This approach used four wavenumbers: 1,504, 1,620, 1,647 and 1,728 cm^−1^ (Fig. 6A; see ESI Table S7), as determined by the min cost of function G (Fig. 6B). GA-LDA produced the best separation (Fig. 7C) using 17 variables, selected at the cost function minimum point (Fig. 7B). These were: 1,049, 1,053, 1,253, 1,415, 1,419, 1,423, 1,500, 1,504, 1,512, 1,516, 1,519, 1,527, 1,531, 1,535, 1,539, 1,543 and 1,546 cm^−1^ (Fig. 7A; see ESI Table S8). This separation is also significant (*P* < 0.05).

### Raman spectral dataset

Figure 2D shows the pre-processed Raman spectra. Each colour represents a different category based on the year of collection. Similar to ATR-FTIR spectra, discrimination of categories requires reduction of the complexity of the spectral dataset. Therefore, PCA-LDA, SPA-LDA and GA-LDA were applied to segregate prostate tissues based on their Raman spectra. The PCA-LDA models (Fig. 2E), using six PC scores accounting for >90% of variance, did not reveal any substantial separation (although *P* < 0.05) and there is a large degree of overlap between all categories. The first six wavenumbers responsible for separation were identified by the associated loadings curve. They include 1,418, 1,457, 1,576, 1,657, 1,704 and 1,739 cm^−1^ (Fig. 2F; see ESI Table S9).

Figure 3F shows the SPA-LDA derived scores plot. This approach also exhibited limited segregation of the categories. The cost function minimum point was obtained at 17 wavenumbers (Fig. 3E). These include: 1,000, 1,001, 1,004, 1,062, 1,109, 1,244, 1,294, 1,295, 1,306, 1,336, 1,373, 1,376, 1,436, 1,437, 1,451, 1,671 and 1,655 cm^−1^ (Fig. 3D; see ESI Table S10). GA-LDA generated only a slight segregation between categories (Fig. 4F), when 49 selected wavenumbers were used, as directed by the cost function minimum point (Fig. 4E): 842, 845, 874, 892, 920, 946, 965, 967, 971, 997, 998, 1,010, 1,022, 1,067, 1,087, 1,168, 1,182, 1,185, 1,201, 1,251, 1,265, 1,271, 1,272, 1,310, 1,342, 1,373, 1,405, 1,421, 1,423, 1,457, 1,483, 1,496, 1,499, 1,507, 1,518, 1,560, 1,575, 1,629, 1,652, 1657, 1,660, 1,666, 1,673, 1,700, 1,710, 1,729, 1,733, 1,741 and 1,745 cm^−1^ (Fig. 4D; see ESI Table S11). There is a slight improvement in separation in comparison with PCA-LDA and SPA-LDA approaches (*P* < 0.05).

Analysis of the Raman dataset for categories: 1983–1984 and 2012–2013 by the application of PCA-LDA, SPA-LDA and GA-LDA identified between-category segregation. PCA-LDA using the first six PCs revealed significant separation (*P* < 0.0001) (Fig. 5E). The derived loadings plot shows the main segregating wavenumbers: 1,419, 1,459, 1,567, 1,654 and 1,742 cm^−1^ (Fig. 5F). ESI Table S12 lists their tentative assignments. SPA-LDA using three wavenumbers, as directed by the minimum cost of function G (Fig. 6E): 891, 1,001 and 1,295 cm^−1^ (Fig. 6D; see ESI Table S13), also revealed between-category segregation (Fig. 6F) (*P* < 0.05). GA-LDA of the same dataset generated similar results (Fig. 7F), which are also statistically significant (*P* < 0.05). In this case, 14 variables were used, at the minimum cost function point (Fig. 7E): 861, 899, 920, 921, 971, 1,049, 1100, 1,204, 1,206, 1,261, 1,365, 1,447, 1,496 and 1,596 cm^−1^ (Fig. 7D; see ESI Table S14).

### Immunochemistry

**Figure 8:**
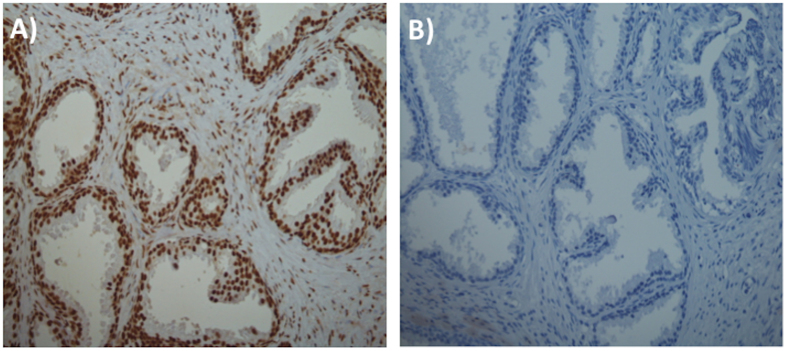
Immunohistochemistry of specimens for genomic 5-methyl cytosine. (**A**) Typical specimen from the 1983–1984 category showing intense global methylation. (**B**) Specimens from the 2012–2013 category showing negative staining for global methylation. Methylation was scored in all cell types.

To further evaluate potential epigenetic changes contributing to trans-generational variability, we performed immunohistochemistry (Fig. 8) in the form of methylation studies. The 1983–1984 (*n* = 10) and 2012–2013 (*n* = 10) categories were compared blindly by a consultant histopathologist. Methylation was graded according to the intensity of staining from 3 (Fig. 8A) to 0 (Fig. 8B) (0 = no staining, 1 = weak staining, 2 = moderate staining, 3 = strong staining). The percentage of cells exhibiting the particular grade within different cellular compartments (epithelial, basal, stromal and vascular cells) was also recorded (see ESI, Table S15). The 1983–1984 category exhibited global methylation with 8 specimens displaying strong (3) and 2 moderate staining (2) in nearly 100% of cells for all cellular compartments. Six specimens from the 2012–2013 category exhibited strong staining with 4 exhibiting staining classed as moderate.

## Discussion

This study aimed to identify spectral differences between benign prostate tissues acquired following TURP procedures carried out over the last 30 y on similarly-aged men. Such spectral differences could be the first evidence of phenotypic alterations from one generation to the next. A total of *n* = 156 tissues were analysed using ATR-FTIR and Raman spectroscopy. The specific prostate histological area examined was the transition zone as this is the tissue region excised at TURP^36^,^37^. About 75% of PCa originates in the peripheral zone, which is located postero-laterally to the urethra (Fig. 1)^38^. Some 25% of PCa also arises in the transition zone and these behave differently to peripheral zone cancers, both morphologically and functionally^39^. Micro-environmental cellular communication plays a significant role in cancer initiation and progression^40^; therefore examination of any part of the prostate may provide information that may lead to better understanding disease pre-disposing alterations, *e.g*., prostatic intraepithelial neoplasia^41^.

IR spectra were obtained from the mid-IR region from 900 to 1,800 cm^−1^ as most bio-molecular spectral signatures reside within this area^42^. Raman spectra used contained wavenumbers from 750 to 1,500 cm^−1^ for the same reason^43^. Computational analysis allowed discrimination of prostate tissue according to the year of surgery. The rationale for this approach was to determine if a trans-generational change in the spectral phenotype of this tissue might be detectable. There was apparent separation between the clusters of different categories that became more pronounced as the period between sample collections became larger.

These three computational methods applied to the spectra obtained by both Raman or ATR-FTIR spectroscopy had varying degrees of success in correctly classifying the specimens into categories. For the ATR-FTIR spectral dataset, the weakest approach for classification was PCA-LDA with 49.9% of the population data correctly classified. Six PCs were used as they provided enough variance (>90%) without introducing unwanted noise and therefore arbitrary separation. The related scores plot (Fig. 2B) shows co-clustering between some of the categories, but also separation between the 1983–1984 and 2012–2013 categories.

GA-LDA was the best method for classification of the ATR-FTIR dataset with 92.3% of the sample correctly classified. SPA-LDA ranked second for classification proficiency (84.2%). Both approaches revealed segregation and a temporal progression between the different categories. Interestingly, both chemometric approaches identified a shift where the “1983–1984” category cluster is completely segregated from the “2012–2013” one.

The Raman spectral data analysis also revealed significant segregation between the different categories. The different chemometric methods had varying success rates in correctly classifying the data. PCA-LDA and SPA-LDA correctly classified 35.8%, while GA-LDA correctly classified 38.6% of the sample population. Despite its weaker classification attainment, Raman spectroscopy pointed to spectral regions representing similar biochemical entities to ATR-FTIR spectroscopy; for example, Amide I, Amide III, collagen and more importantly, changes involving DNA/RNA nucleotide bases and backbone. The markedly reduced variability exhibited by Raman spectroscopy in comparison to ATR-FTIR may be due to the area of tissue interrogated for the acquisition of each spectrum with each technique. The larger surface area sampled by the ATR probe (≈250 μm × 250 μm) has an averaging effect which in this case may be advantageous as it delivers information on the biochemical signature over multiples of cells within the same histological region. Raman on the other hand acquires spectra from a much smaller area and therefore is affected more by micro topographical variations. Nevertheless, the two techniques are potentially complementary, highlighting variability within similar biomolecular regions.

The hypothesis that the chemo-molecular make-up of the prostate gland has changed within one generation is supported by the biospectroscopic techniques employed in this study. The prostate tissues used originated from procedures to treat BPH, which is influenced by nutritional variations including alcohol, vegetables and red meat^44^,^45^. BPH also has potential causal relationships with features of metabolic syndromes like diabetes, hypertension, obesity, high insulin and low HDL-cholesterol^46^,^47^,^48^. These relationships may be determined by genetic or epigenetic events that develop due to hormone-driven events or chemical exposures causing the formation of DNA adducts^49^,^50^. Both ATR-FTIR and Raman spectral analysis highlighted marked trans-generational variation in the spectral regions containing DNA and RNA bands (≈1,000–1,490 cm^−1^) involving nucleic acids, phosphate and deoxyribose modifications. This may point towards alterations that have occurred through chemical genotoxicity or through epigenetic modification of chromatin structure^51^. Also interesting is that SPA and GA algorithms identified wavenumbers indicating variability within the protein region involving amino acid conformational changes in C-O, C-H and N-H. This could be due to post-translational modifications related to genetic and/or epigenetic changes evident within the DNA/RNA spectral regions. Interestingly, the featured spectral areas may point towards a genetic or epigenetic alterations with the variation becoming more pronounced as the period between sample acquisitions increases. Although the small population analysed by immunohistochemistry does not allow for statistically significant results, more specimens from the 1983–1984 category showed intense methylation than from the 2012–2013 class. Global demethylation of the genome in parallel with CGI hypermethylation of particular genes with tumour-suppressor function associated with progression to PCa^52^.

This study was performed using prostate tissues taken from TURP procedures. Although H&E parallel sections of the tissue blocks used for spectroscopy did not show any complicating diathermy effect, this might also need to be taken into account. We tried to select a homogeneous population for our sampling. All men were between 60 and 69 y old. Age is the most important predictor of PCa and its incidence rate increases sharply from 144/100,000 to 500/100,000 for men over the age of 65 y^53^. We sampled a population that varied by 10 y in age in order to increase our sample size. The related confounding variability may have affected our results.

All samples in our study were free from PCa. Approximately 10 to 20% of TURP procedures result in the incidental detection of invasive disease^54^. Therefore a big portion of individuals with “silent” PCa may have been excluded from the tested sample. The main limitation of the study is the lack of information regarding the actual lifestyle of our cohort. We unfortunately could not retrieve information on body mass index, weight, diet and alcohol consumption for all individuals. Also, we could not retrieve from their notes, relative comorbidities, *e.g*., diabetes or hypertension. What we wanted to test though was if there is any variability within prostate tissue with time of tissue collection independent of other variables; therefore, knowing associated risk factors may have caused the introduction of unwanted bias to our study. A significant concern would be the quality of the FFPE blocks over time; previous investigations have shown no significant difference between macromolecules extracted from blocks stored over 11–12 y, 5–7 y, or 1–2 y in comparison to current-year blocks^55^. Our observation of a higher level of methylation staining in the older cohort points to the stability of important macromolecules such as DNA.

## Conclusion

Prostate-related population diversity has not been significantly addressed to date. With this study we attempted to discover spectroscopic alterations that would classify prostate tissue from TURP procedures for BPH according to the year the operation was undertaken. We endeavoured to identify prostate variability that may be related to lifestyle changes that have happened within one generation^13^,^14^,^15^.

Utilising two spectroscopic technologies coupled with three chemometric techniques, we observed significant discrimination of the prostate samples according to their year of collection. Also evident was complete segregation of the prostate tissues collected from two different generations nearly 30 y apart as well as progression through the years. Lifestyle changes during the studied generation have been extensively documented. Their association with changes in prostate tissue from individuals suffering from BPH is indicated by our study.

More extensive research in this field is required to assess the ability of vibrational spectroscopy to identify the existence of variations in prostate tissue with time. A study that extends over several generations, say from the 1920s to the present, may unearth further alterations in the biochemical composition of the prostate gland. These alterations may harbour biomarkers associated with the increase in PCa incidence linked to a Westernised lifestyle adaptation. This in turn may assist the identification of lifestyle adjustments for the prevention of PCa.

## Additional Information

**How to cite this article**: Theophilou, G. *et al*. A biospectroscopic analysis of human prostate tissue obtained from different time periods points to a trans-generational alteration in spectral phenotype. *Sci. Rep*. **5**, 13465; doi: 10.1038/srep13465 (2015).

## Supplementary Material

Supplementary Information
