# Corrigendum: A biospectroscopic analysis of human prostate tissue obtained from different time periods points to a trans-generational alteration in spectral phenotype

**DOI:** 10.1038/srep14886

**Published:** 2015-10-16

**Authors:** Georgios Theophilou, Kássio M. G. Lima, Matthew Briggs, Pierre L. Martin-Hirsch, Helen F. Stringfellow, Francis L. Martin

Scientific Reports 5: Article number: 1346510.1038/srep13465; published online: 08272015; updated: 10162015

In the Supplementary Information file originally published with this Article, Table S15 was truncated. This error has been corrected in the Supplementary Information that now accompanies the Article.

