# Supplementary material for: A biospectroscopic analysis of human prostate tissue obtained from different time periods points to a trans-generational alteration in spectral phenotype
Source: Sci Rep. 2015 Aug 27;5:13465. doi: 10.1038/srep13465 (PMC4550877; doi:10.1038/srep13465)
Supplement: Supplementary Information [file srep13465-s1.pdf]

Electronic supplementary information

A biospectroscopic analysis of human prostate tissue obtained from different time periods points to a trans-generational alteration in spectral phenotype

Georgios Theophilou[1, 2], Kássio M.G. Lima[1, 3], Matthew Briggs[2], Pierre L. Martin Hirsch[1, 2], Helen F. Stringfellow[2], Francis L. Martin[1*]

[1]*Centre for Biophotonics, LEC, Lancaster University, Lancaster LA1 4YQ, UK*

[2]*Department of Obstetrics and Gynaecology, Central Lancashire Teaching Hospitals NHS Foundation Trust, Preston, UK*

[3]*Institute of Chemistry, Biological Chemistry and Chemometrics, Federal University of Rio Grande do Norte, Natal 59072-970, RN-Brazil*

[*]**Corresponding Author:** Prof Francis L Martin PhD, Centre for Biophotonics, LEC, Lancaster University, Lancaster LA1, 4YQ, UK; Tel.: +44(0) 1524 510206; Email: f.martin@lancaster.ac.uk

No. of Pages = 17

No. of Tables = 15

Table S1: Number of training, validation and prediction specimens (or spectra) in each year of collections for prostate tissues from the **FTIR** spectral data.

Year of collection	Set Training	Validation	Prediction
1983-1984	**140**	**30**	**30**
1988-1989	**170**	**40**	**40**
1993-1994	**141**	**30**	**30**
1998-1999	**120**	**30**	**30**
2003-2004	**150**	**30**	**30**
2008-2009	**140**	**30**	**30**
2012-2013	**144**	**32**	**32**

Table S2: Number of training, validation and prediction specimens (or spectra) in each year of collections for prostate tissues from the **Raman** spectral data.

Year of collection	Set Training	Validation	Prediction
1983-1984	**130**	**30**	**30**
1988-1989	**170**	**40**	**40**
1993-1994	**140**	**30**	**30**
1998-1999	**129**	**25**	**25**
2003-2004	**148**	**30**	**30**
2008-2009	**140**	**30**	**30**
2012-2013	**150**	**30**	**30**

Table S3: Principal segregating wavenumbers for all categories derived from the loadings (LD1) curve associated with **PCA-LDA** of the **ATR-FTIR** spectral dataset.

**Wavelength (cm^{-1})**	**Biological fingerprint**
1227	PO_2^- asymmetric (phosphate I)
1400	Symmetric stretching vibration of COO^- group of fatty acids and amino acids
1574	C=N adenine
1624	Peak of nucleic acids due to the base carbonyl stretching and ring breathing mode
1674	Unassigned band
1720	C=O

Table S4: Selected wavelengths for **SPA-LDA** analysis for the **ATR-FTIR** dataset for all 7 classes. The model calculated that 23 wavelengths were needed for correct segregation without the introduction of noise.

**Wavelength (cm^{-1})**	**Biological fingerprint**
968	Symmetric stretching mode of dianionic phosphate monoesters of phosphorylated proteins or cellular nucleic acids, DNA
1018	ν(CO), ν(CC), δ(OCH), ring (polysaccharides, pectin)
1053	ν_sCO-O-C-C-O stretching coupled with C-O bending of the C-OH of carbohydrates, Glycogen
1153	Stretching vibrations of hydrogen-bonding, C-OH groups
1234	Composed of Amide III as well as phosphate vibrations of nucleic acids, $CH_{'6, 20\ a,a'}$ rock
1315	Amide III band components of collagen
1392	Carbon particle
1415	Deformation C-H, N-H, stretching C-N
1446	δ(CH2), lipids, fatty acids δ(CH) (polysaccharides, pectin)
1462	Paraffin
1489	In-plane CH bending vibration
1512	In-plane CH bending, vibrations from the phenyl rings
1539	Protein Amide II absorption: predominately β-sheet of Amide II
1562	Ring base
1593	C=N, NH_2 adenine
1620	Peak of nucleic acids due to the base carbonyl stretching and ring breathing mode
1631	Amide I region
1651	Amide I region
1666	C_5O stretching vibrations of pyrimidine base
1693	A high frequency vibration of an antiparallel β-sheet of Amide I (the Amide I band is due to in-plane stretching of the C=O band weakly coupled to stretching of the C-N and in-plane bending of the N-H bond)
1716	C=O thymine, Amide I (arises from C=O stretching vibration), C=O stretching vibrations of DNA and RNA, C=O stretching vibrations of purine bases
1735	C=O stretching (lipids)
1797	Lipids

Table S5: Selected wavelengths for **GA-LDA** analysis for the **ATR-FTIR** dataset for all 7 classes. The model calculated that 32 wavelengths were needed for correct segregation without the introduction of noise.

**Wavelength (cm^{-1})**	**Biological fingerprint**
987	OCH_3 (polysaccharides-cellulose)
999	Ring stretching vibrations mixed strongly with CH in-plane bending
1002	Unassigned band
1026	Carbohydrates peak for solutions, Vibrational frequency of CH_2OH groups of carbohydrates (including glucose, fructose, glycogen, etc.), Glycogen
1029	O-CH_3 stretching of methoxy groups
1072	Phosphate I band for two different C-O vibrations of deoxyribose in DNA in disordering structure
1191	Deoxyribose
1199	Collagen, Phosphate (P=O) band
1299	Deformation N-H cytosine
1303	Unassigned band
1350	Unassigned band
1353	Unassigned band
1365	Stretching C-O, deformation C-H, deformation N-H
1373	Stretching C-N cytosine, guanine
1381	**δ**CH3, Stretching C-O, deformation C-H, deformation N-H
1388	Carbon particle
1392	Unassigned band
1404	CH_3 asymmetric deformation
1415	Deformation C-H, N-H, stretching C-N
1458	$\boldsymbol{\delta}_{as}CH_3$ of collagen
1496	C=C, deformation C-H
1504	In-plane CH bending vibration from the phenyl rings
1512	In-plane CH bending vibration from the phenyl rings
1543	Amide II
1554	Ring base
1562	Ring base
1589	Ring C-C stretch of phenyl
1600	C=N cytosine, N-H adenine
1647	Amide I in normal tissues: for cancer, is in lower frequencies
1708	C=O thymine
1720	C=O
1751	ν(C=C) lipids, fatty acids

Table S6: Principal segregating wavenumbers for categories: 1983-1984 and 2012-1013 derived from the loadings (LD1) curve associated with **PCA-LDA** of the **ATR-FTIR** spectral dataset.

**Wavelength (cm^{-1})**	**Biological fingerprint**
1231	Overlapping of the protein Amide III and the nucleic acid phosphate vibrations
1400	Symmetric stretching vibrations of COO^- group of fatty acids and amino acids
1447	Asymmetric CH_3 bending of the methyl groups of proteins
1578	Ring C-C stretch of phenyl
1624	Peak of nucleic acids due to the base carbonyl stretching and ring breathing mode
1674	Unassigned band

Table S7: Selected wavelengths for **SPA-LDA** analysis for the **ATR-FTIR** dataset for classes 1983-1984 and 2012-1013. The model calculated that 4 wavelengths were needed for correct segregation without the introduction of noise.

**Wavelength (cm^{-1})**	**Biochemical fingerprint**
1504	In-plane CH bending vibration from the phenyl rings
1620	Peak of nucleic acids due to the base carbonyl stretching and ring breathing mode
1647	Amide I in normal tissues-for cancer is in lower frequencies
1728	C=O band

Table S8: Selected wavelengths for **GA-LDA** analysis for the **ATR-FTIR** dataset for classes 1983-1984 and 2012-2013. The model calculated that 17 wavelengths were needed for correct segregation without the introduction of noise.

**Wavelength (cm^{-1})**	**Biochemical fingerprint**
1049	C-O stretching coupled with C-O bending of the C-OH of carbohydrates, Glycogen
1053	νC-O & δC-O of carbohydrates, Shoulder of 1121 cm^{-1} band, due to DNA
1253	Not assigned
1415	Deformation C-H, N-H, stretching C-N
1423	Not assigned
1500	In-plane CH bending vibration from the phenyl rings
1504	In-plane CH bending vibration from the phenyl rings
1512	Not assigned
1516	Amide II
1519	Not assigned
1527	Stretching C=N, C=C
1531	Modified guanine
1535	Stretching C=N, C=C
1539	Not assigned
1543	Amide II
1546	Amide II (δN-H, νC-N)

Table S9: Principal segregating wavenumbers for all categories derived from the loadings (LD1) curve associated with **PCA-LDA** of the **Raman** spectral dataset.

**Wavelength (cm^{-1})**	**Biological fingerprint**
1418	CH_2 scissoring vibration (lipid band)
1457	Deoxyribose
1576	Nucleic acid mode
1657	Triglycerides (fatty acids)
1704	C=O stretching vibrations of cortisone
1739	Ester group

Table S10: Selected wavelengths for **SPA-LDA** analysis for the **Raman** dataset for all 7 classes. The model calculated that 17 wavelengths were needed for correct segregation without the introduction of noise.

**Wavelength (cm^{-1})**	**Biochemical fingerprint**
1000	Phenylalanine, Bound & free NADH
1001	Phenylalanine
1004	Phenylalanine (of collagen), ν_s(C-C), symmetric ring breathing, phenylalanine (protein assignment)
1062	C-C skeletal stretch random conformation
1109	Benzoid ring deformation
1244	Amide III
1294	Methylene twisting
1295	Methylene twisting
1306	CH_3/CH_2 twisting or bending mode of lipid/collagen, CH_3/CH_2 twisting, wagging &/or bending mode of collagens & lipids
1336	Polynucleotide chain (DNA purine bases), δ(CH3), δ(CH_2) twisting, collagen (protein assignment)
1373	T, A, G (ring breathing modes of the DNA/RNA bases)
1376	Unassigned band
1436	CH_2 scissoring
1437	CH_2 deformation
14551	CH_2CH_3 deformation
1655	Amide I
1671	Amide I

Table S11: Selected wavelengths for **GA-LDA** analysis for the **Raman** dataset for all 7 classes. The model calculated that 49 wavelengths were needed for correct segregation without the introduction of noise.

**Wavelength (cm^{-1})**	**Biochemical fingerprint**
842	Glucose
845	Unassigned band
874	C-C stretching, hypro (collagen assignment)
892	Backbone, C-C skeletal
920	C-C stretch of proline ring/glucose/lactic acid C-C, proline ring (collagen assignment)
946	Unassigned band
965	Hydroxyapatite
967	Lipids
971	ν(C-C) wagging
997	C-O ribose, C-C
998	ν45(CC), observed in the spectra of single human RBC
1010	Unassigned band
1022	Glycogen
1067	Proline (collagen assignment)
1087	ν1CO232, n3PO342, ν(C-C) skeletal of acyl back-bone in lipid (gauche conformation)
1168	Lipids ν(C=C) δ(COH) (lipid assignment) n(C-C), carotenoid
1182	Cytosine, guanine, adenine
1185	Anti-symmetric phosphate vibrations
1201	Nucleic acids and phosphates Aromatic C-O and C-N
1251	Guanine, cytosine (NH_2)
1265	Amide III
1271	Amide III
1310	CH_3/CH_2 twisting, wagging &/or bending mode of collagens & lipids
1342	G (DNA/RNA), CH deformation (proteins and carbohydrates)
1373	T, A, G (ring breathing modes of the DNA/RNA bases)
1405	$\nu_s COO_2$ (IgG)
1421	A, G (ring breathing modes of the DNA/RNA bases)
1423	NH in-plane deformation
1457	Deoxyribose
1483	Unassigned band
1496	Unassigned band
1499	C=C stretching in benzenoid ring
1507	Cytosine
1518	ν(C=C), porphyrin, Carotenoid peaks due to C-C & conjugated C_5C band stretch
1560	Tryptophan
1575	Ring breathing modes in the DNA bases G, A (ring breathing modes

	of the DNA/RNA bases)
1629	Ca=Ca stretch, Amide C=O stretching absorption for the β-form polypeptide films
1652	Lipid (C=C stretch)
1657	Fatty acids<br>Amide I (collagen assignment)<br>Triglycerides (fatty acids)
1600	Amide I
1666	Collagen
1673	Amide I
1700	ν(C=O)OH (amino acids aspartic & glutamic acid)
1710	One of absorption positions for the C=O stretching vibrations of cortisone
1729	Ester group
1733	One of absorption positions for the C=O stretching vibrations
1741	Ester group
1745	ν(C=O), phospholipids, Triglycerides (fatty acids), ν(C=O) (polysaccharides, pectin)

Table S12: Principal segregating wavenumbers for categories: 1983-1984 and 2012-1013 derived from the loadings (LD1) curve associated with **PCA-LDA** of the **Raman** spectral dataset.

**Wavenumber (cm^{-1})**	**Biological fingerprint**
1419	Ester group
1459	Deoxyribose, $\delta(CH_2)$
1567	Unassigned band
1654	C=C stretch & the Amide I bands, Amide I
1709	C=O stretching vibrations of cortisone
1742	Ester group

Table S13: Selected wavelengths for **SPA-LDA** analysis for the **Raman** dataset for classes 1983-1984 and 2012-2013. The model calculated that 3 wavelengths were needed for correct segregation without the introduction of noise.

**Wavelength (cm^{-1})**	**Biochemical fingerprint**
891	Saccharide band (overlaps with acyl band)
1001	Symmetric ring breathing mode of phenylalanine
1295	Not assigned

Table S14: Selected wavelengths for **GA-LDA** analysis for the **Raman** dataset for classes 1983-1984 and 2012-2013. The model calculated that 14 wavelengths were needed for correct segregation without the introduction of noise.

**Wavelength (cm^{-1})**	**Biochemical fingerprint**
861	Phosphate group
899	Monosaccharides (β-glucose), (C-O-C) skeletal mode
920	C-C stretch of proline ring/glucose/lactic acid C-C,<br>praline ring (collagen assignment)
921	Not assigned
971	ν(C-C) wagging
1049	Glycogen
1100	C-C vibration mode of the gauche-bonded chain
1204	Amide III & CH_2 wagging vibrations from glycine backbone & proline side-chains, collagen
1206	Hydroxyproline, tyrosine (collagen assignment)
1261	Not assigned
1365	Tryptophan
1447	CH_2 bending mode of proteins & lipids
1496	Not assigned
1596	Not assigned

Table S15. Methylation studies for classes of 1983-1984 and 2012-2013. An intensity score from 0 to 3 was given to each cellular type along with the percentage of cells showing that intensity.

Class/ Sample		Epithelial cells	Basal cells	Stromal cells	Vascular cells
1983-1984 1	Percentage %	100	100	100	100
	Intensity score	3	3	3	3
1983-1984 2	Percentage %	100	100	100	100
	Intensity score	3	3	3	3
1983-1984 3	Percentage %	100	100	100	100
	Intensity score	3	3	3	3
1983-1984 4	Percentage %	100	100	100	100
	Intensity score	3	3	3	3
1983-1984 5	Percentage %	100	100	100	100
	Intensity score	3	3	3	3
1983-1984 6	Percentage %	100	100	100	100
	Intensity score	3	3	3	3
1983-1984 7	Percentage %	100	100	100	100
	Intensity score	3	3	3	3
